# Parathyroid Gland Response to Vitamin D Deficiency in Type 2 Diabetes Mellitus: An Observational Study

**DOI:** 10.7759/cureus.3656

**Published:** 2018-11-28

**Authors:** Azhar Hussain, Omar B Latiwesh, Alia Ali, Elsa Tabrez, Lalit Mehra, Fidelis Nwachukwu

**Affiliations:** 1 Epidemiology and Public Health, Xavier University School of Medicine, Oranjestad, ABW; 2 Pathology, Higher Institute of Medical Professions, Benghazi, LBY; 3 Internal Medicine, Sheikh Zayed Hospital, Lahore, PAK; 4 Internal Medicine, American University of Integrative Sciences, Bridgetown, BRB; 5 Anatomy and Histology, Xavier University School of Medicine, Oranjestad, ABW; 6 Pathology, Xavier University School of Medicine, Oranjestad, ABW

**Keywords:** vitamin d deficiency, insufficiency, diabetes mellitus, insulin, parathyroid

## Abstract

Background

Studies have linked vitamin D deficiency with the risk of type 2 diabetes mellitus (T2DM) and to the development of chronic complication of diabetes. Vitamin D receptors (VDR) have been found in many tissues in the body including the pancreas, a finding that indicates its role in insulin secretion. In addition, many studies have demonstrated the role of vitamin D and its receptor in insulin sensitivity and signal transduction. Vitamin D deficiency is common throughout the world, but not all vitamin D deficiencies are accompanied by a rise in parathyroid hormone (PTH). The present study was conducted to assess vitamin D deficiency in type 2 diabetic patients in comparison to healthy control and to determine parathyroid gland response to vitamin D deficiency in both groups.

Methods

This observational study was performed during a period from January to October 2018. The study included 151 type 2 diabetic patients selected from three diabetes clinics and 43 age and sex-matched healthy subjects. Informed consent and clinical information were obtained from all participants before the study. Results of the laboratory analysis for serum 25-hydroxyvitamin D (25-OHD), PTH, calcium, and phosphorous were recorded. The data was analyzed using the statistical package for the social sciences (SPSS) Statistics 17.

Results

The results showed low vitamin D concentration in both groups; however, there was no significant difference in vitamin D concentration between diabetic patients and the control patients. A high percentage of PTH level was found in severe vitamin D deficient diabetic patients and healthy controls. The higher percentage of diabetic and normal subjects with mild vitamin D deficiency had a normal PTH level. All healthy subjects with vitamin D insufficiency showed normal PTH concentration. About 10% of diabetic patients with severe vitamin D deficiency had a low PTH level.

Conclusion

The population in our study was generally deficient in 25-OHD irrespective of diabetes mellitus, indicating a greater need for vitamin D supplementation. Not all vitamin D deficient patients have high PTH levels, a finding that supports the emergence of new criteria for vitamin D deficiency, diagnosis and treatment, and highlights the importance of testing PTH in this regard.

## Introduction

Hyperparathyroidism is a disease that occurs due to increased secretion of parathyroid hormone (mainly PTH) from parathyroid glands and as a result, causes hypercalcemia [1]. Secondary hyperparathyroidism is a response to low calcium levels related to hypovitaminosis D. It is well established that there is an inverse relationship between serum 25-hydroxyvitamin D (25-OHD) and serum PTH [2]. Not all vitamin D deficient individuals manifest with increased PTH levels; this response being dependent upon the severity of vitamin D deficiency. Vitamin D deficiency can be categorized into three stages. Stage 1 being vitamin D insufficiency (mild), stage 2 being vitamin D deficiency (moderate), and stage 3 being the more severe form of vitamin D deficiency. Patients are assigned a stage depending on their serum 25-OHD (nmol/L) and percentage of serum PTH increase. Stage 1 individuals have serum 25-OHD of 25–50 nmol/L and 15% increase in serum PTH. Stage 2 individuals have serum 25-OHD < 25 nmol/L and 15–30% increase in serum PTH. Stage 3 individuals have serum 25-OHD < 12.5 nmol/L and >30% increase in serum PTH.

The levels of 25-OHD that lead to a rise in serum PTH are still a matter of debate [3]. Reports suggest that not all who are vitamin D insufficient have increased PTH levels [4, 5]. It has been suggested that the variability of PTH levels in hypovitaminosis D may be due to concomitant magnesium deficiency [5]. Patel et al. [6] suggested that the glomerular filtration rate is the single most important factor in maintaining PTH levels. Certain studies, while supporting the kidney function hypothesis, felt that body mass index (BMI) may play a role in women by blunting the level of PTH and added that in men, insulin-like growth factor 1 (IGF1), smoking, and testosterone levels may do the same [7]. A diagnosis of secondary hyperparathyroidism, therefore, depends on the measurement of an insufficient or deficient amount of 25-OHD in association with the high levels of PTH. The World Health Organization (WHO) described a serum level of 25-OHD of 20 ng/ml as a deficiency [8], and a level of 30 ng/ml as normal, because, at this level, PTH drops down to normal levels [9]. At present, it is believed that below 30 ng/ml of 25-OHD, the level of PTH should start rising [10].

Several studies have suggested that low levels of vitamin D may play a role in the development of type 2 diabetes mellitus (T2DM). Indeed, in a meta-analysis of 21 studies, the circulating level of 25-OHD was inversely associated with the risk of future T2DM [11]. Previous studies have also reported that increased PTH is associated with insulin resistance and metabolic syndrome. The deterioration of insulin sensitivity and B-cell function has been described in hyper-parathyroid states [12]. Conversely, however, many investigators have questioned the association of vitamin D and PTH with glucose metabolism [13].

A possible explanation for this conflicting evidence is that previous studies have generally evaluated the respective metabolic implications of vitamin D and PTH in isolation rather than considering both hormones together as a reflection of the status of the PTH-vitamin D axis. Moreover, in the case of vitamin D, the 25-OHD concentration that provides maximal PTH suppression is widely variable, suggesting that there is an individual threshold for the serum 25-OHD concentration below which PTH rises [14]. A number of researchers hypothesized that in order for an optimal evaluation of the impact that vitamin D has on glucose metabolism, it is necessary to assess both 25-OHD and PTH in combination. There is a possibility that glucose metabolism may only be largely affected when 25-OHD declines below the threshold. This would, in turn, cause a rise in PTH levels, indicating verifiable vitamin D inadequacy [15].

Aim of the study

The present study, the first of its kind in Libya, was conducted to assess vitamin D deficiency in type 2 diabetic patients in comparison to healthy control. This study was also intended to determine the parathyroid gland’s response to vitamin D deficiency in both of the aforementioned groups.

## Materials and methods

The present observational study was performed during a period of 10 months from January 2018 until October 2018. In 2018, 151 T2DM patients selected from three diabetes clinics (Alhaya clinic, Alrazy clinic, and Alnukbah clinic) and 43 age and sex-matched healthy subjects were included in the study. Informed consent was obtained from all participants before the study. The selection of patients was based on the previous diagnosis of T2DM according to American Diabetes Association (ADA) criteria (glycated hemoglobin (HbA1c) ≥ 6.5%, or fasting plasma glucose level ≥ 126 mg/dL, or 2-h plasma glucose ≥ 200 mg/dL during an oral glucose tolerance test, or the presence of classical symptoms of hyperglycemia (polyuria, nocturia, polydipsia, etc.) and a random plasma glucose ≥ 200 mg/dL) [16].

Clinical information and medical history were obtained by a physician through the review of patient medical files and patients’ interviews. Face to face interviews were based on a questionnaire that included variables such as age, sex, date of the diagnosis, cause of the disease, treatments, and any health problems or prescriptions.

Patients suffering from any disease other than diabetes that could affect their metabolic status and the parameters studied such as malignancy, malabsorption, chronic diarrhea, chronic kidney disease, infection, hypoparathyroidism, primary hyperparathyroidism, acute or chronic inflammation, chronic liver disease, or tuberculosis were excluded from the study. Pregnant and lactating women were also excluded. The medication history was recorded and patients taking vitamin D or nutrient supplementation during the last six months were also excluded.

The control group consisted of healthy subjects with no history of inherited or acquired parathyroid disorders. They were not suffering from any infections, inflammations, or renal disease.

Results of the laboratory analysis for serum 25-OHD, PTH level, calcium, and phosphorus were recorded after informed consent was obtained from all participants. Serum 25-OHD was estimated by Microplate Washer and Reader (LINEAR, Spain), using a commercial enzyme-linked immunosorbent assay (ELISA) kit (Euroimmum company, Germany). Serum PTH was measured using COBAS e 411 (ROCH, Germany). The measurements of serum calcium (Ca^2+^) and serum phosphorus (PO_4_^3-^) were done using an automated routine chemistry analyzer (Roche Cobas Integra 400 plus, Roche Diagnostics Limited, Switzerland) with commercial kits (Roche Cobas packs, Roche Diagnostics Limited, Switzerland) according to the manufacturer's protocol.

A. Serum 25-OH vitamin D estimation

The test principle is based on the competitive ELISA technique. This technique utilizes the anti-vitamin D monoclonal antibody (mAb Anti-25-hydroxyvitamin D) which is coated in microtiter wells. A measured amount of patient serum and standards are added followed with extraction buffer to the microtiter wells to release vitamin D from its binding protein (vitamin D binding protein (DBP)-complex). After the first incubation step, a constant amount of biotinylated 25-hydroxyvitamin D is added which competes with the endogenous serum vitamin D for a limited number of binding sites on the coated anti-vitamin D antibodies.

After incubation, the conjugate consisting streptavidin-horseradish peroxidase (HRP) (Conjugate 2) is added to bound biotin containing the first conjugate. After incubation, the wells are completely washed to remove unbound 25-OHD and a solution of chromogen-substrate is added and incubated for 15 minutes, resulting in the development of a blue color. The color development is stopped with the addition of stop solution, the color changes to yellow and is measured spectrophotometrically at 450 nm wavelength.

a. The color intensity is proportional to the amount of biotinylated 25-OHD present and is inversely related to the amount of endogenous 25-OHD in the test sample. By reference to a series of vitamin D standards assayed in the same way, the concentration of 25-OHD in the unknown sample is quantified.

B. Determination of serum parathyroid hormone level

Test Principle: Electrochemiluminescence

Electrochemiluminescence (ECL) processes are known to occur with numerous molecules including compounds of ruthenium, osmium, rhenium or other elements. ECL is a process in which highly reactive species are generated from stable precursors at the surface of an electrode. These highly reactive species react with one another producing light. The development of ECL immunoassays is based on the use of a ruthenium chelate as a complex for the development of light. The chemiluminescent reactions that lead to the emission of light from the ruthenium complex are initiated electrically rather than chemically. This is achieved by applying a voltage to the immunological complexes (including the ruthenium complex) that are attached to Streptavidin-coated microparticles.

The chemiluminescent PTH assay employs two monoclonal antibodies which together are specific for human PTH. This concept is known as the “Sandwich Principle”.

$\bullet$ In the first step, the patient sample is combined with a reagent containing biotinylated PTH antibody and a ruthenium-labeled PTH specific antibody in an assay cup. During a nine minutes incubation step, antibodies capture the PTH present in the sample and form sandwich complexes.

$\bullet$ In the second step, streptavidin-coated paramagnetic particles are added. During a second nine minutes incubation, the biotinylated antibody attaches to the streptavidin-coated surface of microparticles.

$\bullet$ After the second incubation, the reaction mixture containing the immune complexes is transported into the measuring cell; the immune complexes are magnetically entrapped on the working electrode, but unbound reagent and sample are washed away by the system buffer.

$\bullet$ In the ECL reaction, the conjugate is a ruthenium-based derivative and the chemiluminescent reaction is electrically stimulated to produce light. The amount of light produced is directly proportional to the amount of PTH present in the sample.

C. Estimation of serum calcium (Ca^+2^)

Test Principle

This method is based on the specific binding of Arsenazo III and calcium at an acidic pH of 6.5, with the resulting shift in the absorption wavelength of the complex. The intensity of the chromophore formed is proportional to the concentration of total calcium in the sample.

The color intensity of the dye formed is directly proportional to the calcium concentration. It is determined by measuring the increase in absorbance at 650 ± 20 nm.

D. Estimation of inorganic phosphate

Test Principle

Inorganic phosphate reacts with molybdic acid forming a phosphomolybdic complex. Its subsequent reduction in alkaline medium generates a blue molybdenum color whose intensity is proportional to the amount of phosphorus present in the sample. The color intensity of the dye formed is directly proportional to the phosphorus concentration. It is determined by measuring the increase in absorbance at 740 ± 10 nm.

E. Statistical analysis

The data was analyzed using the statistical package for the social sciences (SPSS version 17). Descriptive statistical analysis to the study data was done and it included frequency by percentage, mean, standard deviation (SD), and minimum and maximum values after. The study participants were divided into patients and control, firstly according to the stages of the vitamin D deficiency, then subsequently depending on PTH response to vitamin D deficiency.

## Results

The age of T2DM patients ranged from 21 to 70 years with mean age and SD of 42.5 and 12.5, respectively. The male to female ratio in the T2DM subjects was 7:4. The age of healthy control subjects ranged from 19 to 67 years with mean age and SD of 47.4 and 12.4, respectively. The male to female ratio in the healthy control subjects was 1:2.

A. Vitamin D

**Figure 1:**
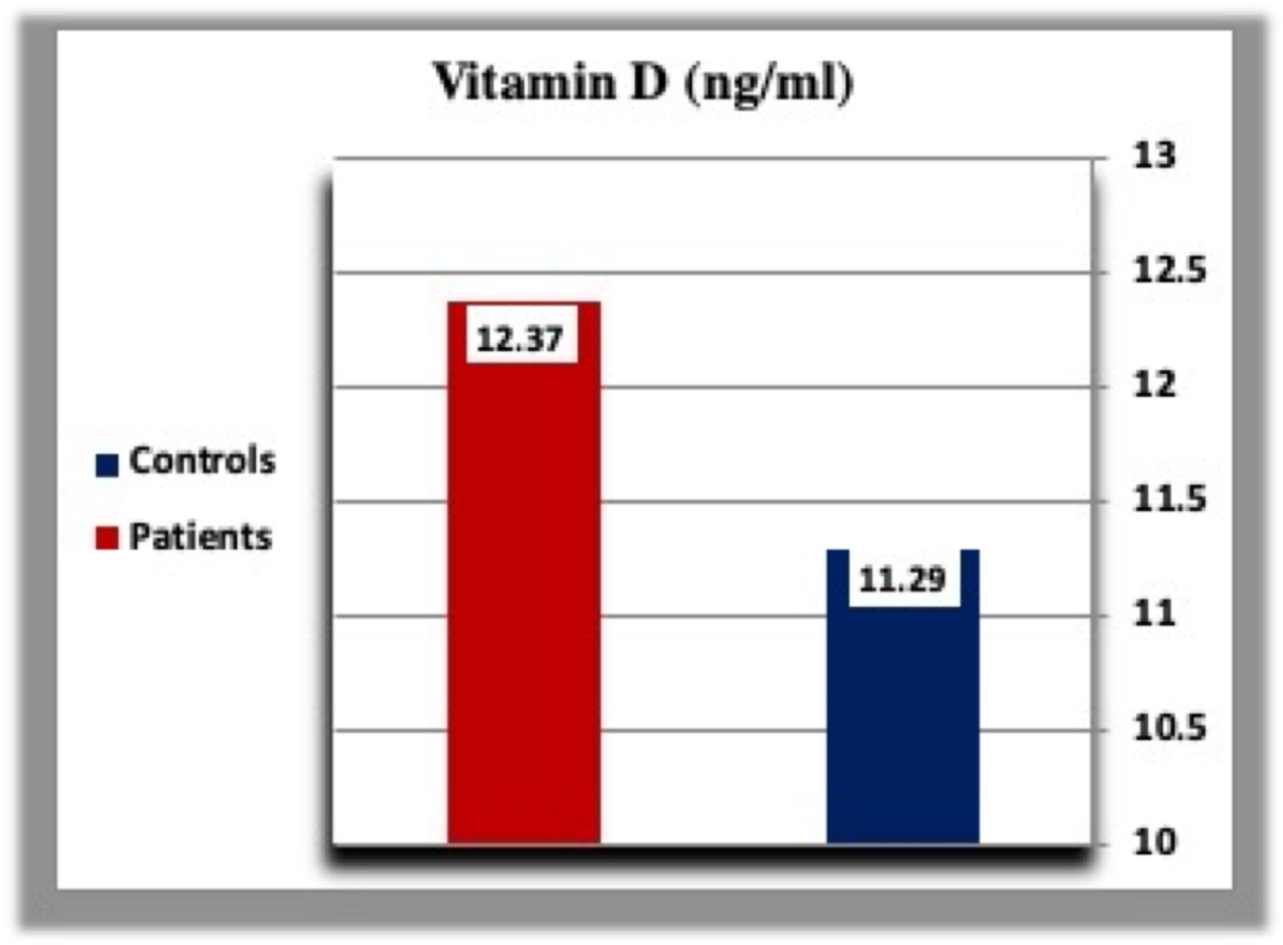
Concentration of vitamin D in diabetic patients and healthy controls.

**Figure 2:**
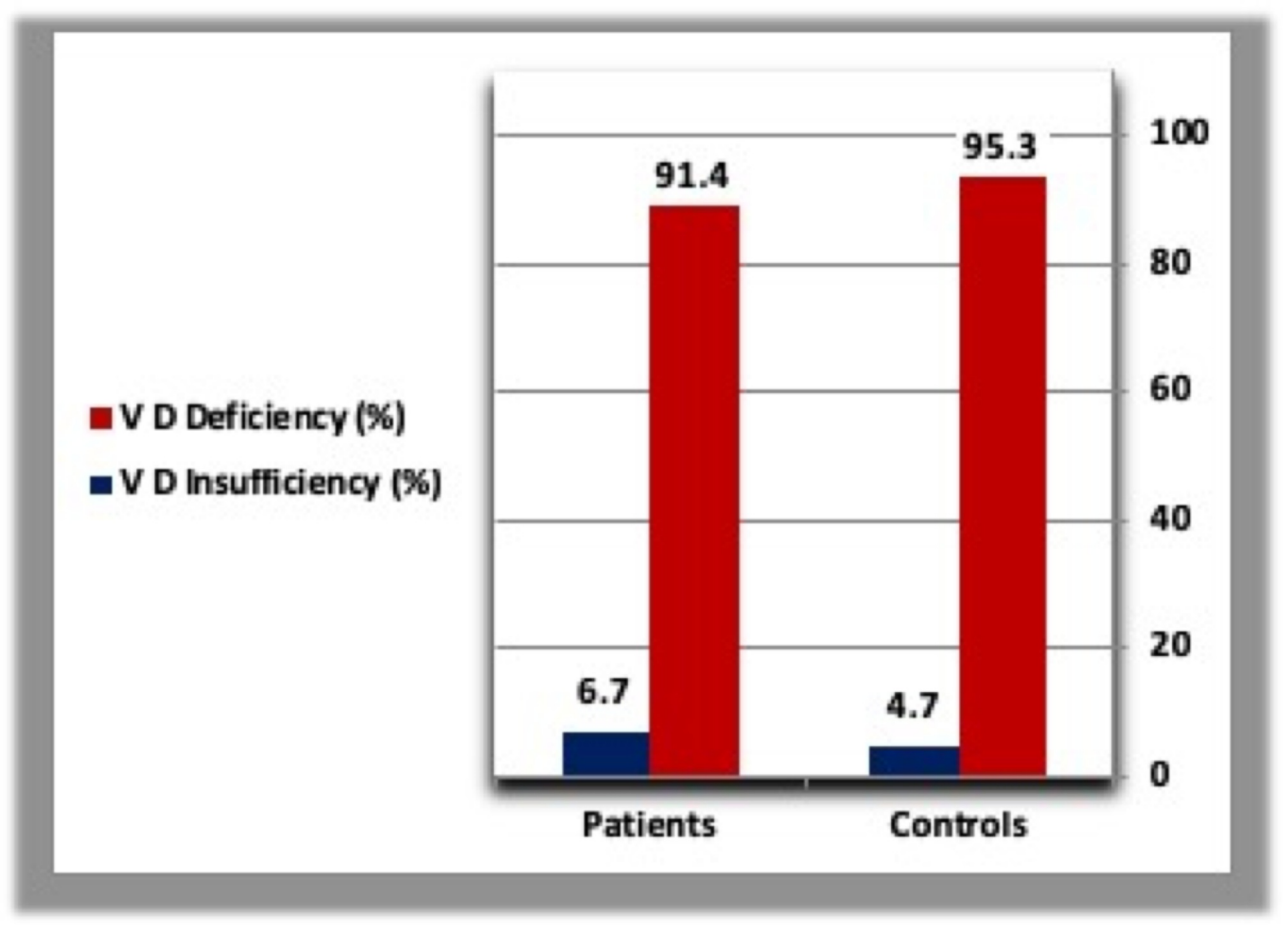
Percentage of diabetic patients and controls with vitamin D deficiency and insufficiency. V D deficiency: Vitamin D Deficiency; V D Insufficiency: Vitamin D Insufficiency.

The mean vitamin D concentration was low in both groups, but there is no statistically significant difference in vitamin D concentration between diabetic patients and controls. Moreover, the percentage of vitamin D deficiency is very high in both T2DM patients and healthy controls (Table 1, Figures 1, 2).

**Table 1:** Vitamin D levels in diabetic patients and healthy controls. N: Number of subjects. Vitamin D deficiency: Vitamin D concentration less than 20 ng/ml. Vitamin D insufficiency: Vitamin D concentration from 29 to 20 ng/ml. *: Percentage.

	Patients (N = 105)	Control (N = 43)
Mean (ng/ml)	12.37	11.29
Standard deviation (SD)	6.17	5.04
Maximum	43.00	29.00
Minimum	Less than 3	4.30
Vitamin D deficiency (%)	91.4	95.3
Vitamin D insufficiency (%)^*^	6.7	4.7

B. Parathyroid hormone, calcium, and phosphorous

**Figure 3:**
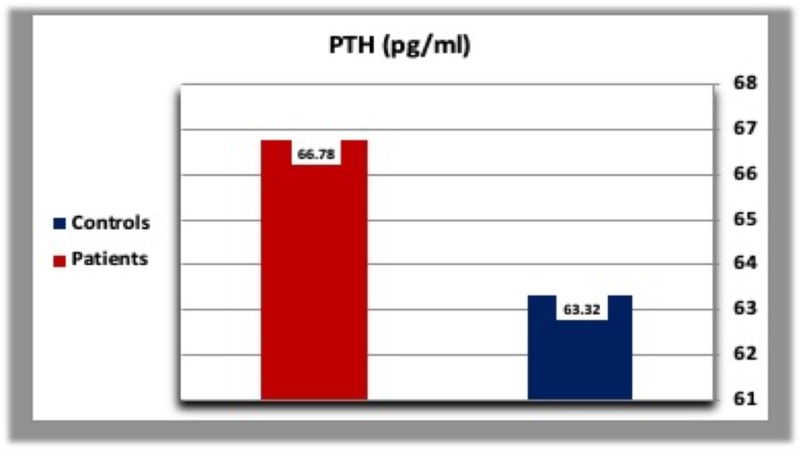
Mean parathyroid hormone concentration in diabetic patients and healthy controls. PTH: Parathyroid hormone

**Figure 4:**
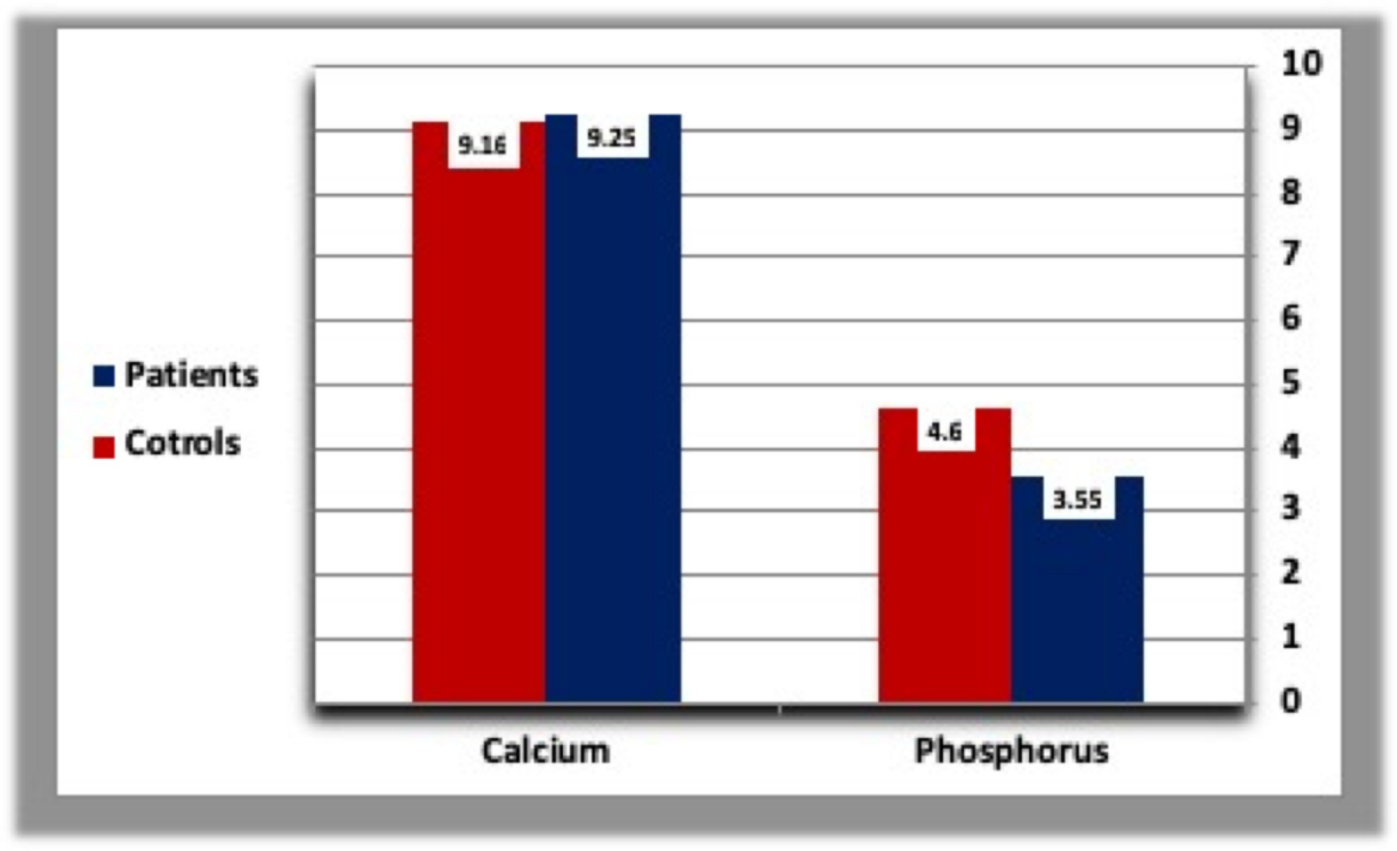
Means calcium concentration (mg/dl) and PO4-concentration in diabetics and normal healthy controls.

There were no significant differences in PTH, calcium, and phosphorous concentrations between T2DM patients and healthy controls (Table 2, Figures 3, 4).

**Table 2:** PTH, calcium, and phosphorous levels in diabetic patients and healthy controls. N: Number of subjects; PTH: Parathyroid hormone.

		PTH (pg/ml)	Calcium (mg/dl)	Phosphorus (mg/dl)
Patients (N = 105)	Mean (mg/dl)	66.78	9.25	3.55
	Standard deviation (SD)	69.65	0.44	0.51
	Maximum	701.00	10.30	4.80
	Minimum	8.40	8.10	2.30
Controls (N = 43)	Mean	63.32	9.16	4.60
	Standard deviation (SD)	33.31	0.43	5.85
	Maximum	157.00	10.10	31.00
	Minimum	26.00	8.40	2.10

C. Categories of vitamin D deficiency

**Figure 5:**
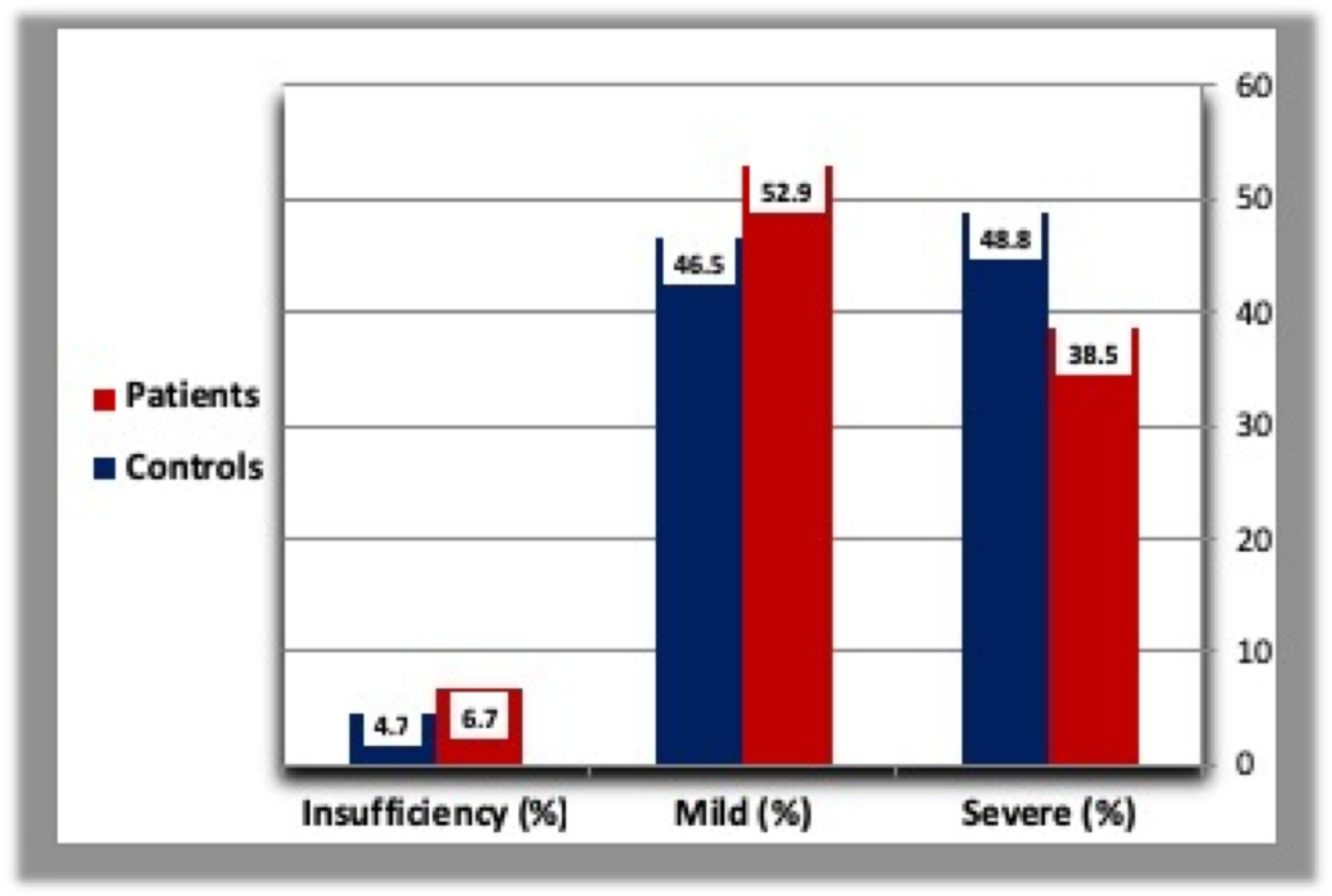
Severity of vitamin D deficiency in diabetic patients and healthy controls.

Mild vitamin D deficiency was most common in T2DM patients followed by severe deficiency. However, severe deficiency was more common than mild deficiency in the control group. A minority of patients (6.7%) and control (4.7%) showed vitamin D insufficiency (Table 3, Figure 5).

**Table 3:** Severity of vitamin D deficiency in diabetic patients and healthy controls. N: Number of subjects. Severe vitamin D deficiency: Vitamin D concentration less than 10 ng/ml. Mild vitamin D deficiency: Vitamin D concentration less than 20 ng/ml. Vitamin D insufficiency: Vitamin D concentration from 29 to 20 ng/ml. *: Percentage.

	Patients (N = 105)	Controls (N = 43)
Severe (%)	38.5	48.8
Mild (%)	52.9	46.5
Insufficiency (%)	6.7	4.7

D. Parathyroid hormone response

**Figure 6:**
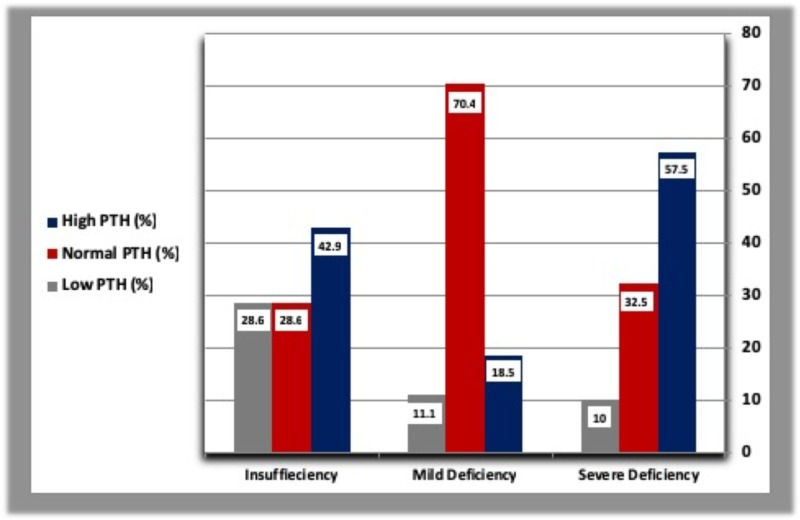
Parathyroid hormone response to vitamin D deficiency in diabetic patients and healthy controls. PTH: Parathyroid hormone

A higher percentage of T2DM patients and healthy controls with severe vitamin D deficiency showed raised PTH levels. The higher percentage of diabetic and healthy subjects with mild vitamin D deficiency had normal PTH level. All of the healthy subjects with vitamin D insufficiency showed normal PTH concentration. Interestingly, about 10% of diabetic patients with severe vitamin D deficiency had low PTH level (Table 4, Figure 6).

**Table 4:** Parathyroid hormone response to vitamin D deficiency in diabetic patients and healthy controls. N: Number of subjects. Low PTH: PTH concentration lower than 30 pg/ml. Normal PTH: PTH concentration within normal range (30–65 pg/ml). High PTH: PTH concentration higher than 65 pg/ml.

		Low PTH	Normal PTH	High PTH
Patients (N = 105)	Severe (%)	10	32.5	57.5
	Mild (%)	11.1	70.4	18.5
	Insufficiency (%)	11.1	28.6	42.9
Controls (N = 43)	Severe (%)	0	38.9	61.1
	Mild (%)	9.1	72.7	18.2
	Insufficiency (%)	0	100.0	0

E. Parathyroid hormone, calcium, and phosphorous levels according to vitamin D deficiency categories

**Figure 7:**
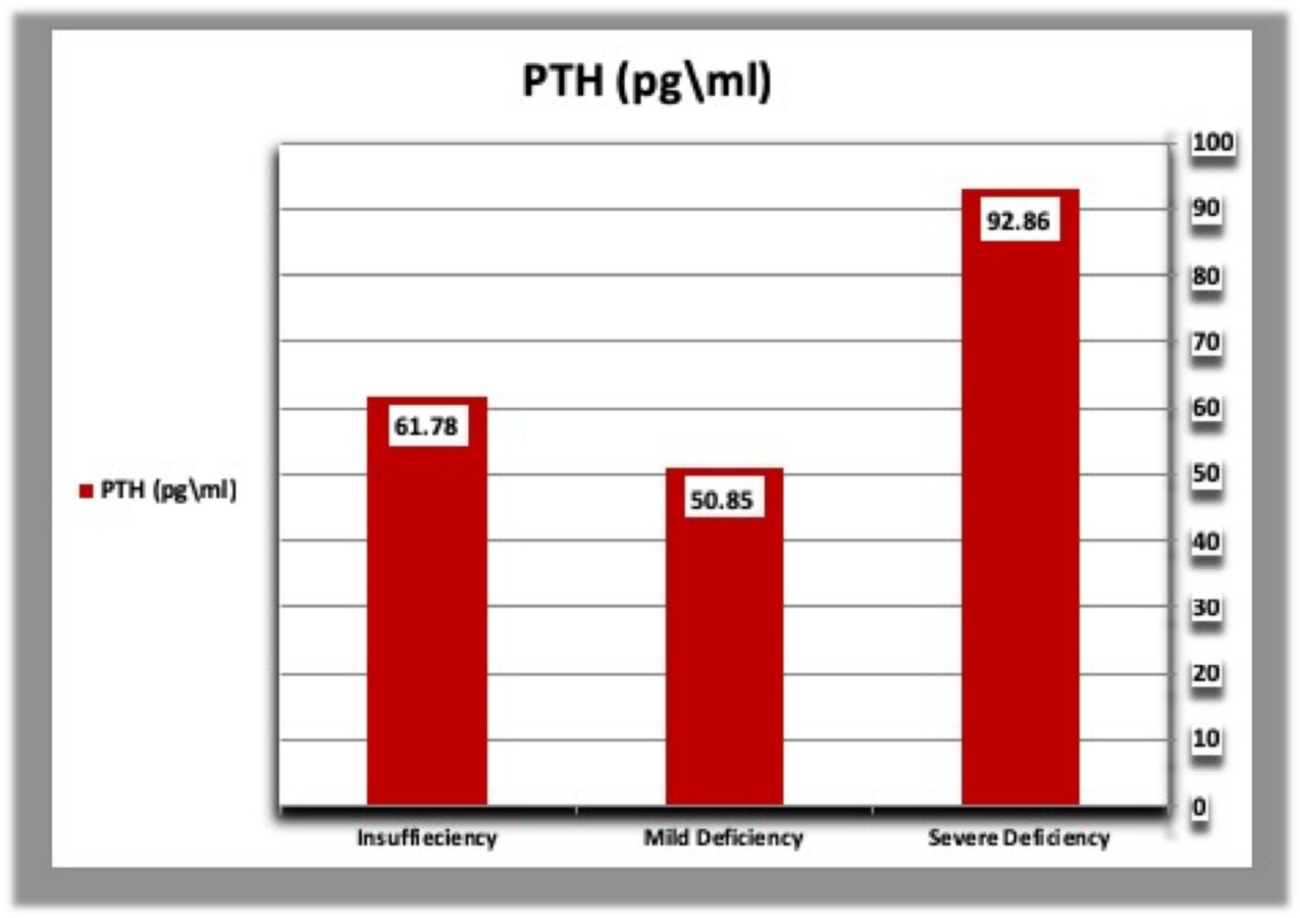
Parathyroid hormone levels according to vitamin D deficiency categories in diabetic patients. PTH: Parathyroid hormone

**Figure 8:**
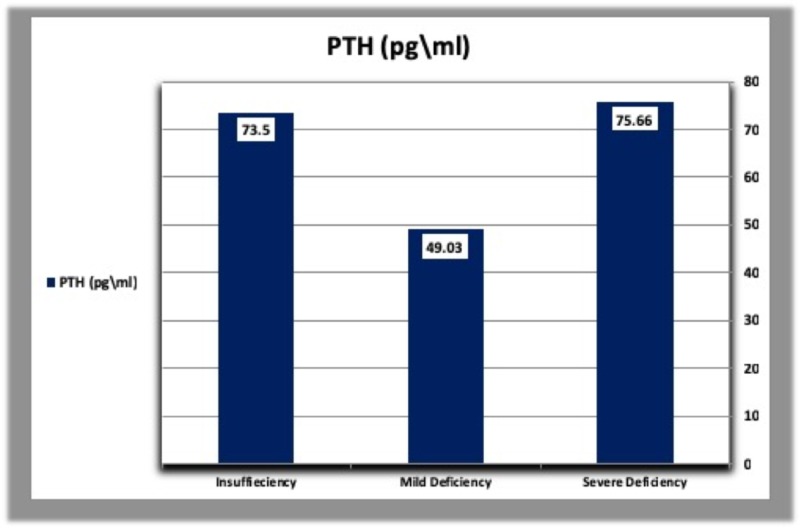
Parathyroid hormone levels according to vitamin D deficiency categories in healthy controls. PTH: Parathyroid hormone

**Figure 9:**
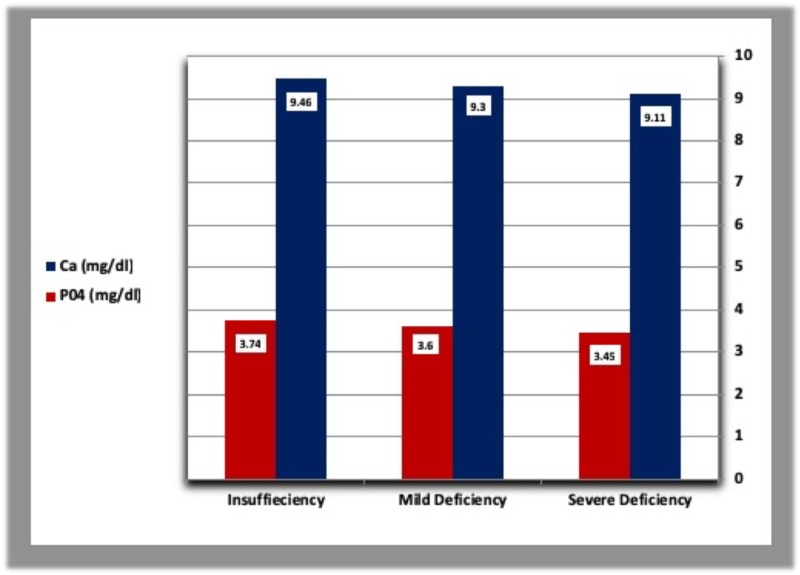
Calcium and phosphorous levels according to vitamin D deficiency categories in diabetic patients.

**Figure 10:**
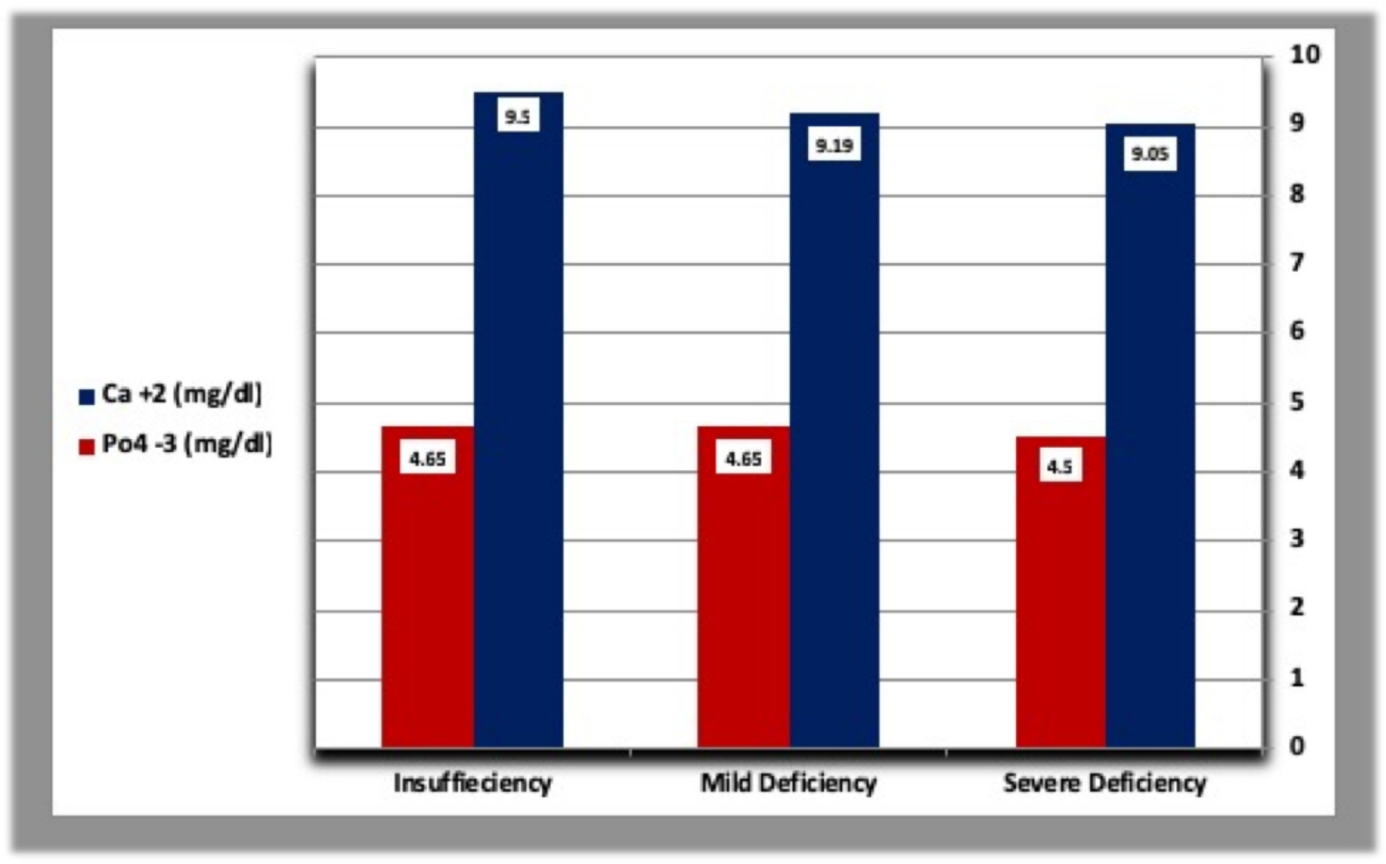
Calcium and phosphorous levels according to vitamin D deficiency categories in healthy controls.

The mean concentration of parathyroid hormone was higher in diabetic patients with severe vitamin D deficiency than patients with mild vitamin D deficiency and those with vitamin D insufficiency. In addition, diabetic patients with vitamin D insufficiency had a higher concentration of PTH than those with mild vitamin D deficiency. In healthy controls, PTH levels were higher in subjects with mild vitamin D deficiency than those with vitamin D insufficiency, but lower than patients with severe vitamin D deficiency (Table 5, Figures 7, 8). The mean calcium level was gradually raised from severe vitamin D deficiency to vitamin D insufficiency in both diabetic patients and controls (Table 5, Figures 9, 10).

**Table 5:** Parathyroid hormone, calcium, and phosphorous levels according to vitamin D deficiency categories in diabetic patients and healthy controls. N: Number of subjects. Severe vitamin D deficiency: Vitamin D concentration less than 10 ng/ml. Mild vitamin D deficiency: Vitamin D concentration less than 20 ng/ml. Vitamin D insufficiency: Vitamin D concentration from 29 to 20 ng/ml.

		Severe	Mild	Insufficiency
Patients (N = 105)	PTH (pg/ml)	92.86	50.85	61.78
	Calcium (mg/dl)	9.11	9.30	9.46
	Phosphorus (mg/dl)	3.45	3.60	3.74
Controls (N = 43)	PTH (pg/ml)	75.66	49.03	37.50
	Calcium (mg/dl)	9.05	9.19	9.50
	Phosphorus (mg/dl)	4.50	4.94	4.65

## Discussion

Vitamin D deficiency has been implicated in decreased insulin secretion and increased insulin resistance, and more recently with the development of T2DM [17]. Due to the presence of 25-OHD insufficiency in up to 91.3% of diabetic patients and 95.4% of healthy controls, any significant difference in vitamin D status could not be ascertained. Alhumaidi et al. reported similar results, where they found that about 98.3% of non-diabetic subjects and about 98.8% of diabetic participants had vitamin D concentration of less than 30 ng/ml. A total of 340 patients (98.5%) from both groups were found to be insufficient in 25-OHD with no considerable difference between the non-diabetic and diabetic groups [18].

Our results revealed a non-significant difference in vitamin D concentration between diabetic patients (12.37 ng/ml), and healthy controls (11.29 ng/ml). One study reported lower serum vitamin D concentration in T2DM patients (20.07 ng/dl), compared to healthy controls (23.98 ng/dl) [19], whereas an alternate study reported a higher vitamin D concentration in diabetics (15.7 ng/ml), compared to healthy controls (11.1 ng/ml) [18].

These findings were explained as insufficient vitamin D level is possibly due to the use of sun protective creams which decrease the penetration of ultraviolet B (UVB) rays into the skin thus preventing the vitamin synthesis. The lack of fortification of food with vitamin D, a diet deficient in vitamin D rich food, as well as a modern lifestyle with little to no sun exposure, could be some of the reasons for deficient/insufficient vitamin D levels.

The effects of vitamin D on glucose metabolism are mainly due to the distribution of its receptors (VDR) on pancreatic β cells, skeletal muscle, and adipose tissue. The presence of 1α hydroxylase in β cells, the presence of vitamin D response element in the human insulin receptor gene promoter also influences insulin sensitivity. Calcitriol directly activates the transcription of human insulin receptor gene, activating peroxisome proliferator activator receptor 𝛿 (PPAR). Vitamin D stimulates the expression of insulin receptor and enhances insulin-mediated glucose transport in vitro [20]. Certain allelic variations in vitamin D receptor gene and DBP might influence glucose tolerance and insulin secretion; thus, contributing to the genetic risk for T2DM [21].

In our study, high PTH levels have been mostly found in severe vitamin D deficiency in both normal and diabetic subjects. Interestingly, about 70% of participants with mild vitamin D deficiency had normal PTH levels, and about 10% of diabetic patients with severe vitamin D deficiency showed low PTH levels.

A study conducted in Saudi Arabia observed that PTH was raised in healthy subjects with vitamin D deficiency by 51.8% as assessed by clinical laboratory improvement amendments (CLIA), 66.23% as assessed by radioimmunoassay (RIA), and 100% as assessed by high-performance liquid chromatography (HPLC) or liquid chromatography-mass spectrometry (LC-MS) [22]. Kilicarslan et al. reported that over 75% of the vitamin D deficient patients had normal levels of PTH [23]; however, in a study by Elsammak et al. [24], PTH did not correlate with serum vitamin D level in either of the genders.

Previous studies were done on healthy subjects of the general population, observing that serum PTH is inversely correlated with 25-OHD [25]. In addition, several studies have reported circulating 25-OHD levels fluctuate between 10 and 50 ng/mL [26]; no threshold has been satisfactorily estimated above which 25-OHD fails to suppress PTH. In most of these studies, serum PTH concentrations begin to decrease as circulating 25-OHD levels rise to 15 to 20 ng/mL and are maximally suppressed at 30 to 40 ng/mL (75 to 100 nmol/L) [27-29]. Few studies also suggest that the relation between 25-OHD and PTH may vary with age and ethnicity [30].

According to the physiological model, vitamin D deficiency results in increased PTH secretion in order to maintain calcium homeostasis. This may result in an increased bone turnover. However, it has been shown that some vitamin D insufficient individuals do not display increased PTH secretion and this has been termed ‘functional hypoparathyroidism’ [4]. Factors other than calcium and vitamin D may modify PTH response, and such factors may vary depending on an individual's sex. For example, IGF1 and testosterone may play a role in men, while smoking, BMI, magnesium level, and kidney function may play a role in women. This may have to be taken into consideration when defining adequate vitamin D status from PTH levels [7].

## Conclusions

The population in our study was generally deficient in 25-OHD irrespective of diabetes mellitus, indicating a greater need for vitamin D supplementation. Not all vitamin D deficient patients have high PTH levels, a finding that supports the emerging of new criteria for vitamin D deficiency, diagnosis and treatment, and highlights the importance of PTH test in this regard.
